# Comparative Transcriptome Analysis of the Heat Stress Response in *Monochamus alternatus* Hope (Coleoptera: Cerambycidae)

**DOI:** 10.3389/fphys.2019.01568

**Published:** 2020-01-21

**Authors:** Hui Li, Xinyi Zhao, Heng Qiao, Xuanyu He, Jiajin Tan, Dejun Hao

**Affiliations:** ^1^Forestry College, Co-Innovation Center for Sustainable Forestry in Southern China, Nanjing Forestry University, Nanjing, China; ^2^Forest Protection, Forestry College, Nanjing Forestry University, Nanjing, China

**Keywords:** heat stress, *Monochamus alternatus*, transcriptome analysis, heat shock proteins, signal transduction, immune system

## Abstract

Temperature is a critical factor of insect population abundance and distribution. *Monochamus alternatus* Hope (Coleoptera: Cerambycidae) is a significant concern since it is transmitted vector of the pinewood nematode posing enormous economic and environmental losses. This pest shows tolerance to heat stress, especially extremely high temperatures. Exposing for 6, 12, 24, 48, or 96 h, the 50% median lethal temperatures (Ltem_50_) for fourth-instar larvae were 47.5, 45.5, 43.9, 43.4, and 42.3°C, respectively. A total of 63,360 unigenes were obtained from complementary DNA libraries of *M. alternatus* fourth-instar larvae (kept at 25°C and exposed to 40°C for 3 h) and annotated with six databases. Five hundred sixty-one genes were significantly upregulated, and 245 genes were downregulated after heat stress. The Gene Ontology enrichment analysis showed that most different expression genes are categorized into “protein folding” and “unfold protein binding” terms. In addition, “Longevity regulating pathway-multiple species,” “Antigen processing and presentation” as well as “MAPK signaling pathway” were significantly enriched Kyoto Encyclopedia of Genes and Genomes pathways. Further analysis of different expression genes showed that metabolism processes were suppressed, while ubiquitin proteolytic system, heat shock proteins, immune response, superoxide dismutase, cytochrome P450s, and aldehyde dehydrogenase were induced after heat shock. The stress signaling transduction pathways such as MAPK, Hippo, and JAK-STAT might be central convergence points in *M. alternatus* heat tolerance mechanism. The expression levels from quantitative real-time PCR of 13 randomly selected genes were consistent with the transcriptome results. These results showed that *M. alternatus* possessed strong heat tolerance and genes related to protein activity, immune response, and signal transduction composed of a complicated heat tolerance mechanism of *M. alternatus*. This research provided new insights into the mechanisms of thermal tolerance in other insects and aided in exploring the function of heat resistance-related genes.

## Introduction

Insects are the most diverse and successful animal population, with essential roles in the terrestrial ecosystems (Foottit and Adler, 2017). The life histories of insects are determined by many abiotic factors, among which temperatures are vital. Temperature can affect the insect population dynamics and geographical distribution by interfering with their development rate and life cycle, as well as the distributions of host plants (Bale et al., 2002). In the face of global warming, frequent extreme temperatures directly or indirectly limit insect survival and affect all physiological and metabolic processes in insects, such as alimentation, digestion, detoxification, mating, growth, and development (Garrad et al., 2016).

Reversely, insects’ geographic distribution is dramatically confined to tolerance ranges to extreme temperature (Jing and Kang, 2004). Researches manifested that 50% median lethal temperature (Ltem_50_) in 24 h of many insects, especially beetles, is over 40°C (Johnson et al., 2004; Li, 2014; Lü and Liu, 2017). Their impressive ability to thrive under high temperature is due to their plastic responses, including behavioral avoidance of extreme temperature by, for example, changing their period of activity within a day or shifting their feeding positions on a plant (Huey, 2002; Kuhrt et al., 2006; Dillon et al., 2009), and physiological adjustments, such as diapause, enhancing their fitness and survival rates under stressful temperature conditions.

There is an increasing interest in investigating insects’ adaptive mechanisms against extreme temperatures. Heat stress can trigger conserved modulating genes, which involved in cellular activities, such as metabolism, protein folding, transport, and degradation (Feder and Hofmann, 1999). Most of the genes encoding cytochrome P450s (P450), antioxidative enzymes, and aldehyde dehydrogenase (ALDH) are significantly upregulated after heat exposure (Wei et al., 2015; Zhang Y.H. et al., 2015; Liu et al., 2017). Constant high-temperature stress is simultaneously capable of inducing genes related to traditional cryoprotectants to protect cells from damage (Wang et al., 2014). Notably, heat shock proteins (HSPs) served as molecular chaperones, reported in *Drosophila melanogaster*, *Glyphodes pyloalis*, and *Anaphothrips obscurus*, are responsible for heat tolerance (Colinet et al., 2013; Liu et al., 2017; Guo and Feng, 2018). In addition, insects could cope with extremely high temperatures by employing immune response and signal transduction (Li et al., 2012; Liu et al., 2017).

*De novo* transcriptome assembly has been widely applied to detect and identify differential genes under different experimental conditions (He et al., 2017; Liu et al., 2017, 2018; Chen et al., 2018), enabling researchers to understand the molecular mechanism of action from a transcriptomics perspective. Different expression gene (DEGs) profiling in this technique presents the advantages of precision, economy, and repeatability, and has been widely used in plants to explore genes related to heat resistance (Li et al., 2015; Yan et al., 2016; Shi et al., 2017), while in insects, comparative transcriptome analysis related to heat responses has been only applied in several species, including *G. pyloalis*, *Cryptolaemus montrouzieri*, and *Bombyx mori* (Wang et al., 2014; Zhang Y.H. et al., 2015; Liu et al., 2017).

The pine sawyer beetle, *Monochamus alternatus* Hope (Coleoptera: Cerambycidae), is the primary vector of the pinewood nematode, *Bursaphelenchus xylophilus* (Steiner et Buhrer) Nickle (Aphelenchida: Parasitaphelenchidae), which is the causative agent of devastating pine wilt disease (Mamiya and Enda, 1972) in China and other East Asian countries. The disease, native to North America, was firstly found in Nanjing City, Jiangsu Province, in 1982, and spread over another 15 provinces by 2018 (Hu and Wu, 2018). The occurrence of pine wilt disease is closely related to the wide distribution of *M. alternatus*, which rests with its strong tolerance to high temperature. As in many parts of China, the maximum temperatures in natural forests often exceed 40°C in summer, and days with temperature over 40°C have been increasing in the last few years (Zhang W. et al., 2015). Therefore, the particular survival mechanism under extremely high temperatures of *M. alternatus* is needed to be clarified. Although a tentative work of *M. alternatus* has ever revealed the upregulation of three *MaHSPs* at 35 and 40°C (Cai et al., 2017), the comprehensive mechanisms of response to heat stress in *M. alternatus* remained to be further explored by transcriptome sequencing.

In the present study, we conducted the bioactivity of Ltem_50_ from 6 to 96 h in *M. alternatus.* We conducted a comparative transcriptomic analysis between *M. alternatus* larvae exposed at normal and high temperatures to identify the significantly upregulated and downregulated genes related to heat tolerance. We performed an analysis of differential expression genes as well as pathways, and qRT-PCR to validate the RNA-seq data. We aimed to provide a basis for the adaptive mechanism of heat tolerance in *M. alternatus* and aided in exploring the function of heat resistance-related genes.

## Materials and Methods

### Insects and Heat Exposure

Second- and third-instar larvae of *M. alternatus* were collected from host trees, *Pinus massoniana*, in Jiujiang city, Jiangxi Province, China, and reared individually on an artificial diet at constant temperature 25°C ± 0.5°C, 60% ± 5% relative humidity in darkness as described by Chen et al. (2017). Three-day old molted fourth-instar larvae reared separately in sterile containers (height: 4.2 cm; diameter: 3.5 cm) were exposed to an environmental chamber within a range of temperatures (35, 40, 42.5, 45, 50°C) and held at the temperature for 6, 12, 24, 48, and 96 h, respectively. In total, 1500 fourth-instar larvae (20 × 5 temperature-treatment × 5 time-treatment × 3 replicate) were used in this study. Following incubation for the desired period, containers were transferred to 25°C for recovery. After 48 h, the survival of *M. alternatus* larvae was counted and those were considered to be dead if no movement was observed when prodded with a dissecting needle (Johnson et al., 2004; Li et al., 2018).

In many parts of China, the summer extreme high temperature (about 40°C) usually lasts for 3–4 h. To perform transcriptomic analysis similar to the natural condition, 3-day-old molted fourth-instar larvae were exposed to 40°C for 3 h as the heat treatment group. Larvae were reared at 25°C as a control group. Each treatment was repeated three times. After the thermal treatment, the three larvae from each group were immediately frozen in liquid nitrogen and stored at −80°C for subsequent experiments.

### RNA Isolation, Library Construction, and Sequencing

Insect stored at −80°C was crushed individually with a mortar and pestle and then transferred to a 2-ml centrifuge tube (Sagon Biotech, China). The total RNA of each sample was isolated with 1.5 ml of Trizol reagent (TaKaRa, Japan) following the manufacturer’s instructions. The amount of total RNA was detected using the NanoDrop 2000 (Termo, Waltham, MA, United States). Potential RNA degradation and contamination was monitored on 1% agarose gels.

Three independent experimental replicates were used for transcriptomic analysis. A total of 1 μg of RNA from the heat-treated and control larvae was supplied to construct the complementary DNA (cDNA) libraries by NEBNext Ultra RNA Library Prep Kits for Illumina (NEB, United States). The mRNA was fragmented and then primed using random hexamers and used as a template for first-strand cDNA synthesis with reverse transcriptase. After purification, cDNA was ligated at the 3′-end with adenine and sequencing adaptors, followed by PCR amplification to create a cDNA library. The cDNA library was then sequenced on the Illumina HiSeq 2000 platform by Shanghai Majorbio Bio-pharm Biotechnology Co. (Shanghai, China). All raw read sequences were deposited in the National Center for Biotechnology Information Short Read Archive database under the accession number of PRJNA548205.

### Assembly and Functional Annotation

Before sequence assembly, adaptor sequences, sequences containing ambiguous “N” nucleotides (with a ratio of “N” > 10%) and low-quality sequences (with quality scores < 20) were removed. The transcriptomes were assembled using the Trinity program (Grabherr et al., 2011). Based on clean reads, *de novo* transcriptome assembly into transcripts without a reference genome was carried out. For homology-based annotation, non-redundant sequences were used to search multiple public databases, including Swiss-Prot^1^, Pfam^2^, non-redundant protein database, Eukaryotic Clusters of Orthologous Groups (COG^3^), Gene Ontology (GO^4^), and Kyoto Encyclopedia of Genes and Genomes (KEGG)^5^.

### Differentially Expressed Gene Analysis

The expression quantity of each gene (fragments per kilobase of exon model per million mapped fragments) was estimated by Cuffdiff software (Trapnell et al., 2012) based on the length of this gene and the counts of reads mapped to this gene. A gene was considered to be differentially expressed when the results from the above tests were all significant at *P*-value < 0.05 (false discovery rate ≤ 0.01) and | log2FC| ≥ 1 by DESeq2. The DEGs were then used for GO and KEGG enrichment analyses. The Goatools^6^ and Perl scripts were used to implement the statistical enrichment of DEGs in the GO and KEGG pathways. *P*-value-corrected < 0.05 was the threshold value for significant enrichment results.

### qRT-PCR Validation

Thirteen genes were randomly selected to quantify the validity of RNA-seq by qRT-PCR in three duplicates. qRT-PCR was performed using cDNA templates of samples from the control and heat treatment groups. cDNA was synthesized from total RNA using the PrimeScript^TM^II 1st Strand cDNA Synthesis Kit (TaKaRa) according to the recommended protocol. To test the integrity of cDNA templates, Ribosomal protein 10 (*RPL10*) was selected as a housekeeping gene (Li et al., 2018). The qRT-PCR primers (Supplementary Table S1) were designed online^7^. The cDNA templates in 10-fold dilution series were used to construct a relative standard curve to determine the PCR efficiency, and all primers reached amplification efficiencies of 95–100%.

qRT-PCR was performed in an Applied Biosystem 7500 System (United States) using SYBR Premix Ex Taq II (TaKaRa), according to the manufacturer’s protocol. The cycling conditions were as follows: (i) 95°C, 30 s; (ii) 95°C, 5 s; (iii) 60°C, 34 s; and (iv) repeat (ii–iii) for 40 cycles. The procedure was followed by an analysis of melting curves ranging from 60°C to 95°C to verify the presence of a single and discrete peak for each reaction product. Each reaction was run in triplicate, and the average threshold cycle (Ct) was calculated for each replicate. The results of qRT-PCRs were analyzed by the 2^–ΔΔCT^ method (Livak and Schmittgen, 2001).

### Statistical Analysis

In heat resistance assays, survival rates were arcsine square-root transformed to normalize variance of the data before analysis. Ltem_50_ values expressed lethal effects of various levels of temperature in a given period. Probit analysis was performed to estimate these values by SPSS 20.0 software (Finney, 1971).

## Results

### Heat Tolerance of *M. alternatus* Fourth-Instar Larvae

**FIGURE 1:**
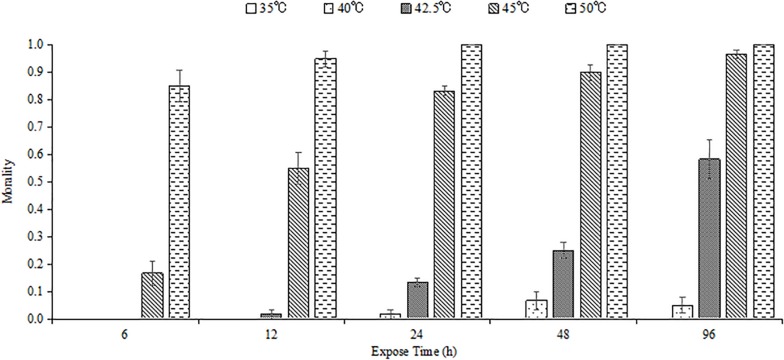
Mean mortality of *M. alternatus* fourth-instar larvae exposed to different high temperatures for 6, 12, 24, 48, and 96 h. Each statistic is the mean of three replicates of 20 larvae per replicate.

High temperature and exposure duration influenced the survival of *M. alternatus* larvae, and mortality increased with enhanced temperature and extended exposure time (Figure 1). Most of the fourth-instar larvae could endure 35 and 40°C for all the tested time. No individual survived when exposed to 50°C for 24 h or longer.

Ltem_50_ values in each experimental time were calculated and are shown in Table 1. Ltem_50_ of *M. alternatus* larvae declined with increasing treatment time in general. When exposed for 6, 12, 24, 48, or 96 h, the Ltem_50_ values for fourth-instar larvae were 47.5, 45.5, 43.9, 43.4, and 42.3°C, respectively.

**TABLE 1:** Fifty percent median lethal temperature (Ltem_50_) for *M. alternatus* fourth-instar larvae when exposed to a range of temperatures for various durations.

**Exposure**	**Ltem_50_ (°C)**	**Slope**	**Intercept**	**Chi-square**	***P*-value**
**time (h)**	**(95% LCL–UCL)**			**(χ^2^)**	
6	47.5 (46.9–48.2)	0.437	−20.772	6.784	0.913
12	45.5 (44.8–46.3)	0.466	−21.196	19.939	0.097
24	43.8 (43.4–44.2)	0.719	−31.497	14.971	0.309
48	43.2 (42.2–44.3)	0.583	−25.178	5.725	0.126
96	42.3 (41.9–42.6)	0.701	−29.616	5.896	0.950

*LCL, lower control limit; UCL, upper control limit.*

### mRNA Sequencing and Assembly

**FIGURE 2:**
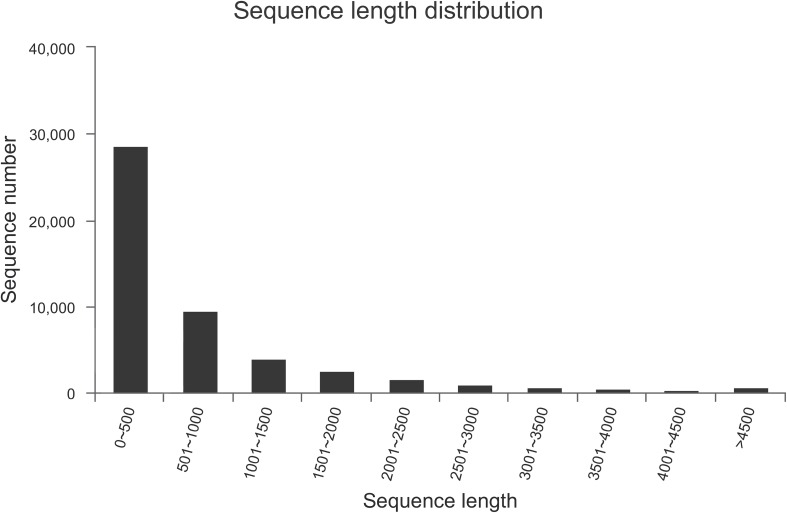
Length and number distribution of unigenes.

We performed transcriptome sequencing to obtain unigenes information of *M. alternatus*, and major characteristics of RNA-seq are summarized in Tables 2, 3. We obtained 14.92 Gb of data and 63,360 unigenes in total. The total and average length of these unigenes were 75,544,544 and 819 bp, respectively, and the N50 was 1590 bp. RNA-seq showed high sequencing quality with 38.70% GC content, over 93 and 97% clean reads of Q20 and Q30, respectively (Tables 2, 3). Unigene length distributions were also determined, with most unigenes being <500 bp (Figure 2). The *M. alternatus* sequences showed 59.05% matches with *Anoplophora glabripennis* (Supplementary Figure S1). We detected 33,060 coding sequences by performing functional annotations.

**TABLE 2:** Quality and base content of RNA-seq.

**Sample**	**Total**	**Total length**	**Mean length**	**N50**	**GC**
	**number**	**(bp)**	**(bp)**		**(%)**
All unigenes	63,360	75,544,544	819	1590	38.70

**TABLE 3:** Distribution and quality metrics of unigenes.

**Sample**	**Control group**	**Heat shock group**
Total raw reads (Mb)	51.79	52.97
Total clean reads (Mb)	50.06	51.38
Total clean bases (Gb)	7.36	7.56
Clean reads Q20 (%)	97.59	97.77
Clean reads Q30 (%)	93.18	93.61
Clean reads ratio (%)^a^	96.66	97.80

*^*a*^Q20/Q30: the ratio of bases, the quality of which is >20/30.*

### Functional Annotation

Moreover, unigenes were annotated with six functional databases described above, and 23,611 (48.21%), 18,257 (37.28%), 17,827 (36.40%), 3647 (7.45%), 13,033 (26.61%), and 13,848 (28.28%) genes were mapped to the six databases, respectively. GO, KEGG, and COG annotation were applied to provide comprehensive functional information for each transcript (Ogata et al., 2000). We annotated 63360 DEGs into three GO categories by GO analysis: biological processes (42.75%), cellular components (45.92%), and molecular function (25.05%). Among which, “cellular process” and “metabolic process” were dominated in the “biological process.” “Cell” and “cell part” were the most representative terms in the “cellular components.” “Binding” and “catalytic activity” were abundant mostly in “molecular function” categories. In addition, 8686 (13.71%) unique transcripts were assigned GO terms from all three categories (Supplementary Table S2 and Supplementary Figure S2). In this study, 19157 unigenes were mapped to 43 secondary pathways. Apart from “Human diseases,” the major pathways in this study were “metabolic pathways” (22.07%). Furthermore, “signal transduction” and “Translation” are the main two secondary pathways (Supplementary Table S3 and Supplementary Figure S3). A total of 1999 unigenes were assigned appropriate COG clusters, which could be classified into 24 functional categories (Supplementary Table S4). Among them, the largest category was “Translation, ribosomal structure and biogenesis” (15.01%); followed by “Posttranslational modification, protein turnover, chaperones” (13.26%).

### Analysis of Differentially Expressed Genes

**FIGURE 3:**
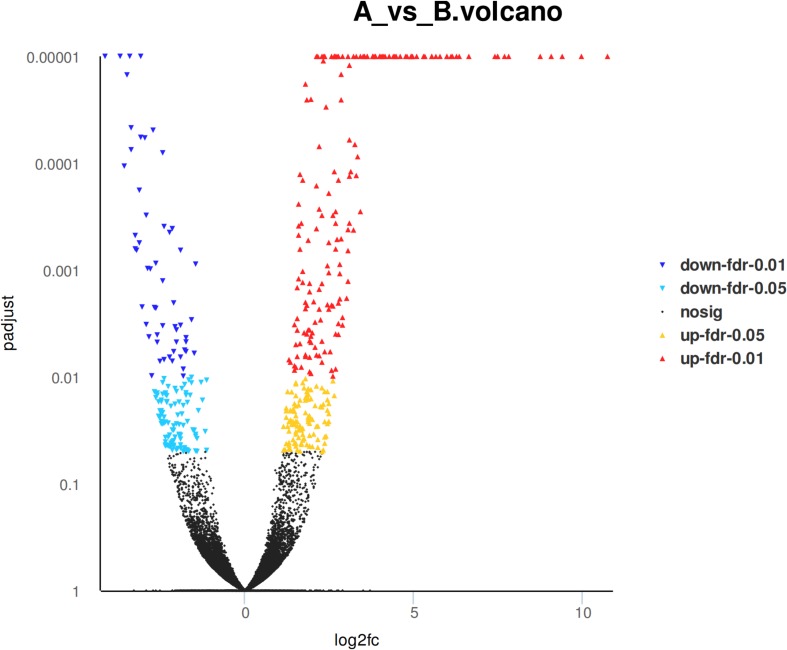
Differentially expressed genes (DEGs) in *M. alternatus* between control (A) and heat shock (B) samples.

**FIGURE 4:**
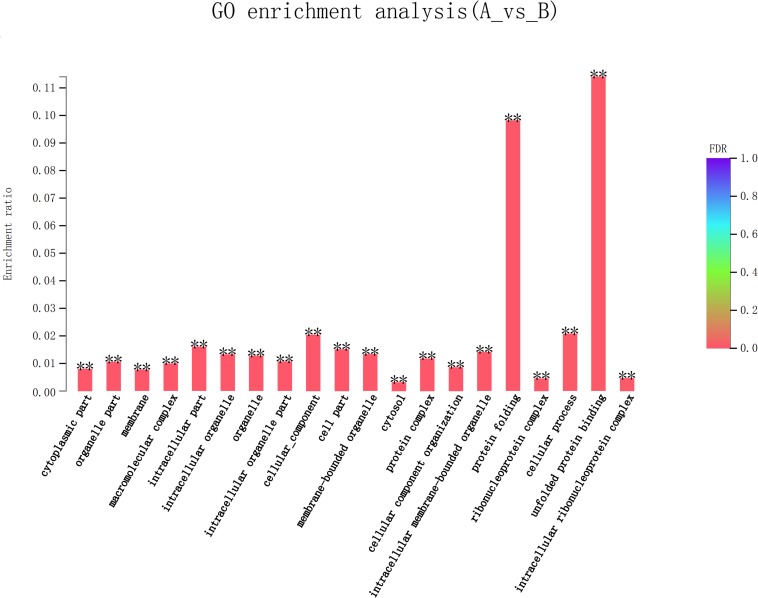
Top 20 Gene Ontology (GO) enrichment analysis of differentially expressed genes (DEGs) between control (A) and heat shock (B) samples. False discovery rate (FDR) < 0.01 was marked as ^∗∗^.

**FIGURE 5:**
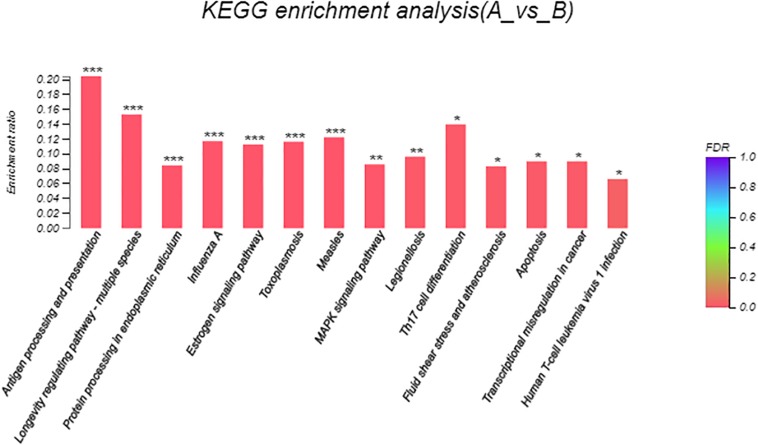
Kyoto Encyclopedia of Genes and Genomes (KEGG) enrichment analysis of differentially expressed genes (DEGs) between control (A) and heat shock (B) samples. FDR < 0.001 was marked as ^∗∗∗^, FDR < 0.01 was marked as ^∗∗^, and FDR < 0.05 was marked as ^∗^.

Eight hundred six unigenes were DEGs after heat shock treatment, with a criterion of *P*-adjust < 0.05 and | log2FC| ≥ 1. There were 545 DEGs upregulated and 261 DEGs downregulated (Figure 3). GO and KEGG enrichment focusing on DEGs could aid in understanding the transcriptional response to heat stress in *M. alternatus*. Figure 4 shows Top20 GO enrichment terms (*P*-value < 0.05), which showed that “protein folding” and “unfold protein binding” are highly enriched. Most of these GO enrichment terms are categorized into “cellular components” and “biological processes.” DEGs (*P*-value < 0.05) were subjected to KEGG enrichment similarly. “Longevity regulating pathway-multiple species” and “Antigen processing and presentation” were the two most significantly enriched KEGG pathways (Figure 5). Notably, the “MAPK signaling pathway” and “Protein processing in endoplasmic reticulum” were also dominant among the significantly enriched pathways.

To further investigate the mechanism in *M. alternatus* responding to heat, we perform further analyses about DEGs involved in GO and KEGG enrichment. Unigenes related to metabolism, protein aggregation, HSPs, immune response, antioxidant, and detoxification, as well as signal transduction were predicted to deal with heat stress in *M. alternatus.*

#### Metabolism Inhibition and Protein Aggregation

**FIGURE 6:**
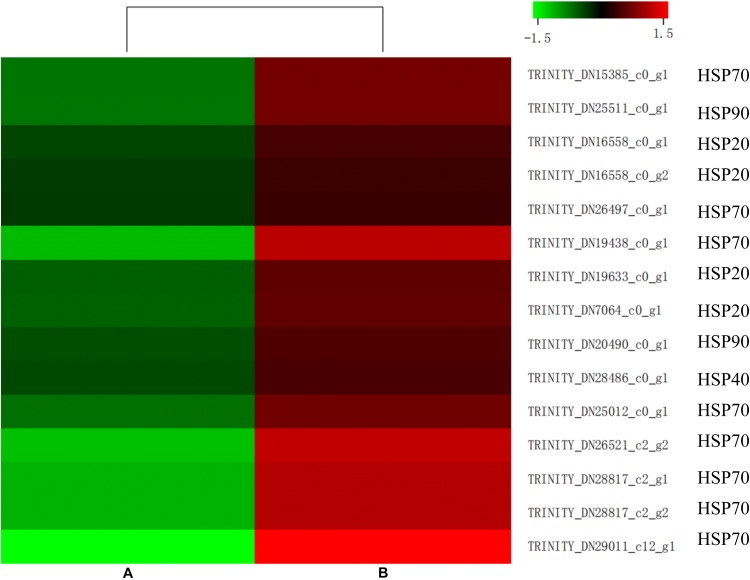
Comparative distribution of heat shock protein (HSP)-coding genes between the control (A) and heat shock (B) groups. The color scale denotes the lowest (green) **(A)** to the highest (red) **(B)** RPKM values.

Some genes related to carbohydrate metabolism were downregulated, such as amino sugar and nucleotide sugar metabolism, showing in Figure 6A. Interestingly, some unigenes associated with misfolding protein turnover processes were upregulated after heat stress treatment. For example, 15 out of 16 genes encoding ubiquitin were significantly induced in the treatment group (Figure 6B).

#### Heat Shock Proteins

**FIGURE 7:**
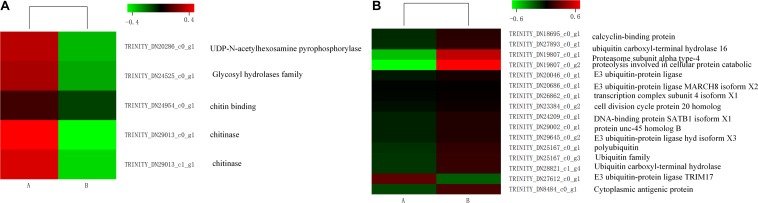
Expression level of genes involved in amino sugar and nucleotide sugar metabolism **(A)** and some genes of ubiquitin **(B)** in control (A) and heat shock (B) samples. The color scale indicates the lowest (green) to the highest (red) RPKM values.

Insects can induce a large number of HSPs after heat exposure, implying that these genes play a vital role in the insect’s molecular mechanism to resist high temperatures (Lang et al., 2009). Our research observed a highly transcriptional level of HSPs after heat stress. HSPs, classified into HSP100, HSP90, HSP70, HSP40, and HSP20 family based on its molecular weight, were upregulated (false discovery rate ≤ 0.001) in the heat-treatment group (Figure 7). Among these 15 induced HSPs, 8 were in the HSP70 family, 4 were part of the HSP20 family, but only 1 and 2 were in the HSP40 and HSP90 families, respectively.

#### Immune Response

**FIGURE 8:**
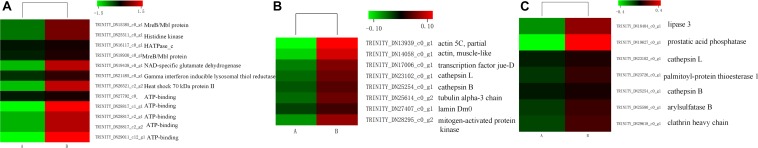
Heatmap of the expression level of antigen processing and presentation **(A)**, apoptosis **(B)**, and genes were involved in lysozymes pathways **(C)** in control (A) and heat shock samples (B). The color scale indicates the lowest (green) to the highest (red) RPKM values.

The RNA-seq analysis revealed that immune-related genes were abundant. A large number of these unigenes were induced in response to heat stress. These genes were mainly involved in pathways related to disease, for example, Epstein–Barr virus infection (13 genes), measles (12 genes), and proteoglycans in cancer (10 genes). Besides, 17 unigenes involved in antigen processing and presentation and 9 unigenes involved in apoptosis were upregulated (Figures 8A,B). Seven genes encoding lysozymes, not belonging to the pathways mentioned above, related to immune defense mechanisms were identified (Figure 8C).

#### Antioxidant and Detoxification

Apart from HSPs, several genes related to antioxidant and detoxification were elicited after heat stress. These genes included one superoxide dismutase (SOD) and three detoxification-related genes, such as genes encoding cytochrome P450s (CYPs) and one gene encoding ALDH (Table 4).

**TABLE 4:** Changes in the transcriptional expression of genes involved in antioxidant and detoxification of *M. alternatus* after exposed to 40°C for 3 h.

**Gene ID**	**Control**	**Heat shock**	**Regulated**	***P-*value**	**Gene description**
TRINITY_DN19531_c0_g1	0.32	1.506666667	Up	0.0050158	[Cu–Zn] SOD
TRINITY_DN25891_c0_g1	20.48666667	99.02666667	Up	1.56E–07	CYP18A1
TRINITY_DN26844_c0_g1	1.986666667	5.003333333	Up	0.001263073	CYP6
TRINITY_DN27264_c2_g2	357.3933333	556.2533333	Up	1.41E–05	CYP6
TRINITY_DN25440_c0_g2	258.4266667	867.65	Up	3.52E–09	ALDH

#### Stress Signal Transduction

Stress signal transduction plays a vital role in insect heat tolerance. Many pathways related to this process were abundant in this study, such as the mitogen-activated protein kinase (MAPK) signaling (13 DEGs), focal adhesion (7 DEGs), Hippo signaling (5 DEGs), PI3K-Akt signaling (5 DEGs), and Janus kinase signal transducer and activator of transcription (JAK-STAT) signaling (4 DEGs) pathways (Supplementary Figure S4).

### Validation of Data Through qRT-PCR

**FIGURE 9:**
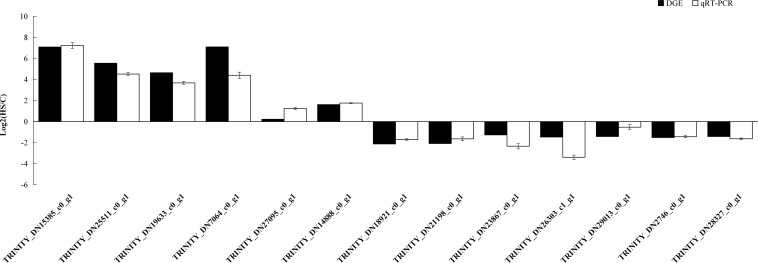
qRT-PCR analysis of the expression levels of 13 unigenes. *X* axis represents the 13 unigenes: TRINITY_DN15385_c0_g1, HSP70; TRINITY_DN25511_c0_g1, HSP70; TRINITY_DN19633_c0_g1, HSP20; TRINITY_DN7064_c0_g1, HSP20; TRINITY_DN27095_c0_g1, glucose dehydrogenase; TRINITY_DN14888 _c0_g1, glyceraldehyde-3-phosphate dehydrogenase; TRINITY_DN18921_c0_g1, general odorant-binding protein; TRINITY_DN21198_c0_g1,beta-1,4-mannosyl-glycoprotein 4-beta-*N*-acetylglucosaminyltransferase TRINITY_DN23867_c0_g1, beta-1,4-*N*-acetylgalactosaminyltransferase bre-4; TRINITY_DN26303_c1_g1, glutathione S-transferase; TRINITY_DN29013_c0_g1, glycosyl hydrolase family 18; TRINITY_DN2746_c0_g1, zinc finger protein Elbow-like; TRINITY_DN28327_c0_g1, multidrug resistance-associated protein. The *Y* axis represents the relative expression levels of genes. *Ribosomal protein10* (*RPL10*) was used as an internal control.

Hundreds of genes showed significantly different expression levels between the control and heat treatment groups. Thirteen genes were randomly selected to evaluate the accuracy of transcriptome sequencing through qRT-PCR. The results showed that expression levels of genes related to heat stress tolerance were upregulated at 40°C. These genes included those encoding HSP70 (TRINITY_DN15385_c0_g1), HSP90 (TRINITY_DN25511_c0_g1), and HSP20 (TRINITY_DN19633_c0_g1 and TRINITY_DN_7064_c0_g_1). We also found that the expression level of *RPL10*, used as an internal control, was stable. The expression levels from qRT-PCR of 13 randomly selected genes were consistent with the DEG expression profiling from transcriptomic results (Figure 9).

## Discussion

High temperatures had adverse effects on insect development and distribution. *M. alternatus* was mainly distributed in the subtropical and tropical regions, which meant that this pest could continue to thrive in continuous hot weather. Accordingly, we first attempted to investigate the resistance of *M. alternatus* larvae against high temperatures. As confirmed by bioassay, the Ltem_50_ of this pest is higher than that of other insects settling in temperature zones. *Xylotrechus rusticus* was a stem-boring pest that mainly occurred in Northeast of China, and the Ltem_50_ of this pest was 36.1°C (33.7–38.1°C) in 96 h largely lower than that of *M. alternatus* (Li, 2014). Similarly, *Frankliniella occidentalis*, originating from western North America, could not survive after exposure to 41°C for 2 h, whereas over 90% *M. alternatus* larvae could survive on 40°C for 96 h. The property of strong heat tolerance in *M. alternatus* evoked great interest from its mechanism responding to high temperature.

RNA-seq could present a comprehensive and accurate gene expression profile for diverse experimental conditions. This technology has been used in *M. alternatus* recently. A previous study had analyzed the RNA-seq data of larval sawyer beetle treated with insecticide (Wu et al., 2016). Here, we detected 33,060 coding sequences in *M. alternatus* and observed 806 DEGs after heat treatment, which could contribute to underlying tolerant events in *M. alternatus.* The specific function of these unigenes could be clarified by six database annotations, especially GO classification, KEGG pathways, and COG terms. Unlike the minor ratio of “cellular component” (28.38%) in previous transcriptome analysis in *M. alternatus* GO annotation (Wu et al., 2016), this category (45.92%) was predominant in our study, which implied that most of the cells required being repaired after heat stress (Howard et al., 2011; Li et al., 2015; Liu et al., 2017). In addition, more KEGG pathways and COG terms related to protein activity and signal transduction were also different from transcriptome data of this beetle treated with insecticide, which all suggested that stress-response mechanisms were diverse in *M. alternatus.*

GO enrichment analysis allowed us to effectively identify critical biological processes that were associated with heat stress response. After heat exposure, top two highly enriched GO terms of “protein folding” and “unfold protein binding” in *M. alternatus* provided evidence for the hypothesis that increasing temperature accelerated protein unfolding and initiated molecular chaperones (Day et al., 2002), similarly confirmed by enrichment of “Protein processing in endoplasmic reticulum” pathways in KEGG analysis. Supposed by enrichment in the “Antigen processing and presentation” pathway, *M. alternatus* might struggle with heat stress by immune response. Transcriptome sequencing in *B. mori* revealed that “longevity regulating pathway–multiple species” pathway was involved in diapause preparation (Chen et al., 2017), whereas this pathway contributed to heat tolerance in our research. The enrichment of the “MAPK signaling pathway” suggested that environmental stress could motivate a signal switch for *M. alternatus*. Enrichment of DEGs manifested that unigenes involved in protein activity, immune response, and signal transduction might be vital components of *M. alternatus* heat-response mechanism. Further analysis of DEGs certified this hypothesis and provided new insights for this mechanism as well.

Metabolizable energy was essential for the maintenance of homeostasis and growth (Richard, 1986). Thermal stress above the optimal temperature harmed the insect’s energy reserves and metabolism (Belhadj et al., 2015). When insects were faced with heat stress, the synthesis of most proteins declines, including those of ATPases participating in three primary metabolisms: glycolytic pathway, tricarboxylic acid cycle, and oxidative phosphorylation. Our analysis of DEGs revealed that many genes related to metabolic processes were repressed. The situation was similar in *G. pyloalis* exposed to 25 and 40°C (Liu et al., 2017). Due to the inhibition of metabolism, protein degradation could be produced when organisms were exposed to heat shock. Heat treatment could induce protein flour hydrolyzates degradation in *Locusta migratoria* and produce low-molecular-weight protein (Purschke et al., 2017). Therefore, some genes performing a function in the removal of damaged proteins would be elicited to maintain cellular structures and activities. The ubiquitin proteolytic system (UPS) had a significant cytoprotective role in degrading damaged proteins (Plafker, 2010). In our study, 15 ubiquitin-related unigenes of *M. alternatus* were upregulated after heat exposure. The results indicated that the UPS could get rid of damaged proteins during insect heat stress.

Similar to ubiquitin, a group of highly conserved proteins, HSPs, could function as molecular chaperones to protect proteins from misfolding and denaturation under heat stress (Montfort et al., 2001; Sun and MacRae, 2005; King and Macrae, 2015). The HSPs were widely distributed in microorganisms, plants, and animals. The large HSP superfamily was commonly classified into several families based on their molecular weight and homologous relationship, including HSP100, HSP90, HSP70, HSP60, HSP40, and HSP20 (Garrido et al., 2012). Since firstly discovered in *D. melanogaster* larvae, HSPs in many insects have already been identified as heat stress-related factors (Ritossa, 1962; Quan et al., 2017; Guo and Feng, 2018; Wang et al., 2019). In the current study, 15 HSPs were upregulated by heat treatment in *M. alternatus*. Four HSP20, known as ATP-independent chaperons of *M. alternatus*, were also induced by heat stress. Function in the first line of cell defense against heat stress, most HSP20 displayed activities in helping the unfolding proteins maintain their correct states, binding to denatured proteins and preventing irreversible protein aggregation when metabolism inhibited (Basha et al., 2012). Interaction between sHsps and Hsp70 is fundamental to the HSP network (King and Macrae, 2015). Hsp70 could remove substrates from sHsps and participates in refolding and degradation, either acting alone or with other HSPs, while this process depends on the energy supplied by the ATPase activity of HSP70. Gene number and expression level of HSP70 were dominant in the induced HSPs, which manifested that HSP70 was the most prominent contributor to thermotolerance in *M. alternatus*. This finding was consistent with the earlier study by Chen et al. (2014) in *Grapholita molesta* and Zhang and Denlinger (2010) in *Helicoverpa zea*. Besides, two HSP90 and an HSP40 were involved in *M. alternatus* heat resistance. HSP90, having similar roles to those of HSP70, could bind a substrate when in an open conformation, but sequestering of proteins is unlikely when ATP is not restrictive, whereas HSP40 could promote substrate binding to HSP70 and enhance ATP hydrolysis to regulate HSP70 activity (Minami et al., 1996; Yonehara et al., 1996). All these results suggested that HSP20, HSP70, and HSP90, in association with HSP40, formed complex molecular networks to boost protein folding and protect cellular proteins from damage in insects.

It was notable that previous studies have certified that HSPs could act as a regulator of immune response (Mehlen et al., 1996; Ravagnan et al., 2001). When blocking HSP70 with special antibody, the production of immune activity (TNF-α released from macrophages) decreased and mammalian HSPs could bind with antigenic peptides (Udono, 1993; Zhou et al., 2018). Therefore, the immune system might be raised by HSPs to cope with heat stress together in *M. alternatus*. In expectation, many unigenes involved in immune response, such as “antigen processing and presentation,” “apoptosis” and “lysozymes,” were exactly induced in the current study. In addition, the homeostasis between cell proliferation and apoptosis were essential for insect survival, while heat stress could activate the excessive occurrence of organisms apoptosis (Takayama et al., 2003; Cui et al., 2016). Practically, the insect immune system was the major effector system to regulate apoptosis. “Antigen processing and presentation” and “lysozymes” were suggested to play a role in the body defense against infection, whereas heat stress might increase disease and impaired longevity; thus, the induction of these genes also certified that the immune response might be evoked directly by heat stress.

In addition to metabolism inhibition and protein degradation, high temperatures could cause oxidative damage, elevating intracellular levels of reactive oxygen species (ROS) that undermined cellular environmental homeostasis and biological functions of some proteins (Monaghan et al., 2009). To prevent damage from ROS, insects have evolved antioxidant defense mechanisms. SOD was the most important antioxidant enzymes in the enzyme defense system against ROS. SOD catalyzed the disputation of superoxide radicals into oxygen (O_2_) and hydrogen peroxide (H_2_O_2_); then H_2_O_2_ was converted by other antioxidant enzymes (catalase and peroxidases) into oxygen and water (H_2_O) (Kang et al., 2017). A previous study found that not only the activity of SOD but also the transcriptional expression of SOD encoding genes were increased significantly when exposed to high temperatures, and the deletion of the SOD gene could produce heat susceptible rice (Pirillo et al., 2010; Chen et al., 2014; Wei et al., 2015; Liu et al., 2017). We detected the upregulation of two SOD genes in the 40°C-treatment *M. alternatus* larvae as well, which revealed that SOD contributed to the ROS-scavenging system in *M*. *alternatus* during heat stress.

Heat stress can induce the production of toxic substances other than oxidative damage. Antioxidant and detoxification mechanisms could work together to cope with heat stress. CYPs catalyzed a broad range of oxidative substances, including insecticides, plant secondary metabolites, and some oxidative substances induced by heat exposure (Daborn et al., 2007; Niu et al., 2011). In the present study, upregulation of CYPs in *M. alternatus*, similar to other organisms treated with heat stress, certified this role of CYPs (Liu et al., 2017; Shi et al., 2017). In addition to CYPs, organisms produced ALDHs in response to a suite of environmental stresses that perturb metabolism, including salinity, dehydration, desiccation, and cold and heat shock (Kirch et al., 2004). The increased expression of genes related to detoxification in *M. alternatus* suggested that CYPs and ALDHs were critical in the oxidative processes derived from heat stress.

Stress-responsive signal transduction pathways could connect the environmental stress with the organism to induce most of these defensive reactions described above (Kawasaki et al., 2002; Ludwig et al., 2005). Sensing the stress signals and transmitting them to cellular machinery to activate adaptive responses are referred to as stress signal transduction (Kawasaki et al., 2002). When suffering from heat stress, organisms could activate various stress-responsive signal transduction pathways, such as PI, Notch, MAPK, Hippo, and JAK-STAT signaling pathways. P38A-MAPK pathways could be activated by diverse stress in mice (Bassi et al., 2014). The activated MAPK could trigger additional signal components to regulate gene expression, cytoskeleton-associated proteins, or enzyme activities, or target certain signal proteins for degradation (Xiong and Zhu, 2001). Therefore the upregulation of heat-responsive genes, including UPS, HSPs, SOD, and CYPs, might be activated by MAPK pathways in *M. alternatus*. JAK-STAT had an important role in the control of immune response, and dysregulation of this signaling was associated with various immune disorders (Shuai and Liu, 2003). Hippo signaling pathway as a central mechanism that regulated tissue homeostasis in species spanning from *Drosophila* to mammals and the dysregulation of Hippo signaling underlies various human diseases (Pan, 2010). The upregulation of these two pathways related to immune response indicated that signal pathways might also pose an immune response in *M. alternatus.* Thus, the stress signaling transduction might be central convergence points in *M. alternatus* heat tolerance mechanism.

The heat tolerance mechanism of *M. alternatus* was complicated and involved lots of genes and pathways. *M. alternatus* could perceive stress signals by MAPK, Hippo, and JAK-STAT signaling pathways and activate heat shock response. UPS and HSPs could protect protein misfolding and get rid of damaging proteins produced by abnormal metabolism and superabundant ROS. Immune response could be either raised by HSPs or induced directly by stress to contribute to this process. In addition, antioxidant and detoxification mechanisms could cooperate to deal with oxidative damage.

## Conclusion

Here, we determined the Ltem_50_ of *M. alternatus* larvae by bioassay to confirm its heat resistance. A total of 63,360 unigenes were obtained, and 806 DEGs were identified in *M. alternatus* after heat stress by RNA-seq. The GO and KEGG pathway enrichment analysis indicated that DEGs participated in the “protein unfolding” and “binding,” “immune response,” and “signal transduction” pathways. Further analysis of 545 upregulated and 261 downregulated DEGs revealed that genes related to metabolism, UPS, HSPs, antioxidants, detoxification, immune response, and signal transduction might work together to defend heat stress in *M. alternatus*, which could be confirmed via more in-depth functional verification experiments. In conclusion, our study provides new insights into the tolerant events underlying heat resistance in *M. alternatus* and other insects, which could contribute to exploring the function of heat resistance-related genes.

## Data Availability Statement

Publicly available datasets were analyzed in this study. This data can be found here: https://www.ncbi.nlm.nih.gov/sra/?term=PRJNA548205.

## Ethics Statement

There was no requirement to seek ethical approval to carry out the work described above. However, the use of insects in the above experiments was kept to a minimum.

## Author Contributions

HL conceived and designed the experiments. HL, XZ, HQ, XH, and JT performed the experiments. HL and DH wrote the manuscript. All authors reviewed the manuscript.

## Conflict of Interest

The authors declare that the research was conducted in the absence of any commercial or financial relationships that could be construed as a potential conflict of interest.

## Footnotes

1 www.ebi.ac.uk/uniprot/

2 http://pfam.xfam.org/

3 www.ncbi.nlm.nih.gov/COG/

4 www.geneontology.org/

5 www.genome.jp/kegg/

6 https://github.com/tanghaibao/GOatools

7 www.genscript.com/tools/real-time-pcr-tagman-primer-design-tool
