# Supplementary material for: Comparative Transcriptome Analysis of the Heat Stress Response in Monochamus alternatus Hope (Coleoptera: Cerambycidae)
Source: Front Physiol. 2020 Jan 21;10:1568. doi: 10.3389/fphys.2019.01568 (PMC6985590; doi:10.3389/fphys.2019.01568)
Supplement: Supplementary file 1 [file Data_Sheet_1.docx]

Supplementary Material

Table S1. Oligonucleotide Primers Used for qRT-PCR validation

| Primer name | Primer sequence (5’→3’) |
| --- | --- |
| TRINITY_DN15385_c0_g1F  TRINITY_DN15385_c0_g1R  TRINITY_DN25511_c0_g1F  TRINITY_DN25511_c0_g1R  TRINITY_DN19633_c0_g1F  TRINITY_DN19633_c0_g1R  TRINITY_DN7064_c0_g1F  TRINITY_DN7064_c0_g1R  TRINITY_DN27095_c0_g1F  TRINITY_DN27095_c0_g1R  TRINITY_DN14888_c0_g1F  TRINITY_DN14888_c0_g1R  TRINITY_DN18921_c0_g1F  TRINITY_DN18921_c0_g1R  TRINITY_DN21198_c0_g1F  TRINITY_DN21198_c0_g1R  TRINITY_DN23867_c0_g1F  TRINITY_DN23867_c0_g1R  TRINITY_DN26303_c1_g1F  TRINITY_DN26303_c1_g1R  TRINITY_DN29013_c0_g1F  TRINITY_DN29013_c0_g1R  TRINITY_DN2746_c0_g1F  TRINITY_DN2746_c0_g1R  TRINITY_DN28327_c0_g1F  TRINITY_DN28327_c0_g1 | AAGCCACAGCTGGTGATACT  GGAAGCTTGTGCTGAGGAAG  TCGTCTCGTCGAATCACCAT  TGGCAGCCATGTAACCCATA  ATTGTCCTCGGATGGAGTTT  GCCTCTTCAGGCACTTCTTG  TGGCTAAGAAGGTTGCTGGA  CGCCCAATTCAGAGCAATCA  CAGAAGGAAGGGCTCTTGGA  TCGTGGAAATGTGCGTCTTC  GCTGATGCTCCAATGTTCGT  TTGGGTAGCGGTGATGGAAT  CGGAGAATTTACCGACGAACC  TCAGGTGGTAGTAGTTGCGT  GATGGCATGGCAATGACTGT  ATCGGCCACGTATTCCGTAT  GAACACTCCTTCTCCTGGCT  TGCATGTGCTGAAGGAACAG  GGAGCTAAGGACTTCATTCCA  CTGCAGCTTCACAAGTCGAT  GCAATCAAGGACAGCTCCAG  TCAATCGGTGGCAGGTAAGT  CTCGCTGCAGCTTCTCATTT  TGTGTAGGAGGAAGCACTGT  GATGTTTCATCACGCGCAAC  AGCTGCTGTTTGACCCAAAG |

**Table S2. Unigenes of *M. alternatus* into three GO categories**

| GO ID (Lev2) | GO Term (Lev2) | Term type | Seq Number |
| --- | --- | --- | --- |
| GO:0000988 | transcription factor activity, protein binding | molecular_function | 87 |
| GO:0005215 | transporter activity | molecular_function | 747 |
| GO:0045735 | nutrient reservoir activity | molecular_function | 4 |
| GO:0001071 | nucleic acid binding transcription factor activity | molecular_function | 216 |
| GO:0045182 | translation regulator activity | molecular_function | 3 |
| GO:0003824 | catalytic activity | molecular_function | 5860 |
| GO:0005488 | binding | molecular_function | 7268 |
| GO:0060089 | molecular transducer activity | molecular_function | 307 |
| GO:0005198 | structural molecule activity | molecular_function | 526 |
| GO:0045499 | chemorepellent activity | molecular_function | 1 |
| GO:0004871 | signal transducer activity | molecular_function | 357 |
| GO:0098772 | molecular function regulator | molecular_function | 317 |
| GO:0009055 | electron carrier activity | molecular_function | 104 |
| GO:0031386 | protein tag | molecular_function | 2 |
| GO:0042056 | chemoattractant activity | molecular_function | 4 |
| GO:0016209 | antioxidant activity | molecular_function | 66 |
| GO:0016530 | metallochaperone activity | molecular_function | 4 |
| Total geneS of MF | | | 15873 |
| GO:0044456 | synapse part | cellular_component | 98 |
| GO:0044421 | extracellular region part | cellular_component | 425 |
| GO:0030054 | cell junction | cellular_component | 180 |
| GO:0044464 | cell part | cellular_component | 5154 |
| GO:0005576 | extracellular region | cellular_component | 592 |
| GO:0005623 | cell | cellular_component | 5231 |
| GO:0032991 | macromolecular complex | cellular_component | 2218 |
| GO:0044422 | organelle part | cellular_component | 2276 |
| GO:0016020 | membrane | cellular_component | 4149 |
| GO:0009295 | nucleoid | cellular_component | 26 |
| GO:0044425 | membrane part | cellular_component | 3701 |
| GO:0044215 | other organism | cellular_component | 5 |
| GO:0099080 | supramolecular complex | cellular_component | 141 |
| GO:0045202 | synapse | cellular_component | 108 |
| GO:0043226 | organelle | cellular_component | 3815 |
| GO:0031974 | membrane-enclosed lumen | cellular_component | 968 |
| GO:0044217 | other organism part | cellular_component | 5 |
| Total geneS of CC | | | 29092 |
| GO:0001906 | cell killing | biological_process | 12 |
| GO:0009987 | cellular process | biological_process | 5914 |
| GO:0008152 | metabolic process | biological_process | 5010 |
| GO:0040007 | growth | biological_process | 85 |
| GO:0048511 | rhythmic process | biological_process | 24 |
| GO:0048519 | negative regulation of biological process | biological_process | 465 |
| GO:0050789 | regulation of biological process | biological_process | 1853 |
| GO:0032501 | multicellular organismal process | biological_process | 607 |
| GO:0051704 | multi-organism process | biological_process | 255 |
| GO:0032502 | developmental process | biological_process | 565 |
| GO:0022610 | biological adhesion | biological_process | 161 |
| GO:0065007 | biological regulation | biological_process | 1986 |
| GO:0000003 | reproduction | biological_process | 156 |
| GO:0051179 | localization | biological_process | 1338 |
| GO:0023052 | signaling | biological_process | 905 |
| GO:0071840 | cellular component organization or biogenesis | biological_process | 1372 |
| GO:0048518 | positive regulation of biological process | biological_process | 560 |
| GO:0098754 | detoxification | biological_process | 5 |
| GO:0050896 | response to stimulus | biological_process | 1445 |
| GO:0099531 | presynaptic process involved in chemical synaptic transmission | biological_process | 14 |
| GO:0022414 | reproductive process | biological_process | 153 |
| GO:0007610 | behavior | biological_process | 31 |
| GO:0002376 | immune system process | biological_process | 249 |
| GO:0040011 | locomotion | biological_process | 129 |
| GO:0044699 | single-organism process | biological_process | 3790 |
| Total genes of BP | | | 27084 |

**Table S3. Unigenes of *M. alternatus* into KEGG pathways**

| First Category | Second Category | num（seq_list） |
| --- | --- | --- |
| Metabolism | Nucleotide metabolism | 444 |
| Metabolism | Biosynthesis of other secondary metabolites | 29 |
| Metabolism | Glycan biosynthesis and metabolism | 285 |
| Metabolism | Metabolism of terpenoids and polyketides | 95 |
| Metabolism | Lipid metabolism | 608 |
| Metabolism | Energy metabolism | 395 |
| Metabolism | Metabolism of other amino acids | 265 |
| Metabolism | Metabolism of cofactors and vitamins | 393 |
| Metabolism | Xenobiotics biodegradation and metabolism | 182 |
| Metabolism | Amino acid metabolism | 652 |
| Metabolism | Carbohydrate metabolism | 880 |
| Total genes of Metabolism | | 4228 |
| Genetic Information Processing | Transcription | 477 |
| Genetic Information Processing | Translation | 1115 |
| Genetic Information Processing | Replication and repair | 262 |
| Genetic Information Processing | Folding, sorting and degradation | 984 |
| Total genes of Information Processing |  | 2838 |
| Environmental Information Processing | Signal transduction | 1507 |
| Environmental Information Processing | Membrane transport | 136 |
| Environmental Information Processing | Signaling molecules and interaction | 182 |
| Total genes of Environmental Information Processing | | 1825 |
| Cellular Processes | Transport and catabolism | 972 |
| Cellular Processes | Cell motility | 174 |
| Cellular Processes | Cell growth and death | 476 |
| Cellular Processes | Cellular community - eukaryotes | 465 |
| Total genes of Cellular Processes | | 2087 |
| Organismal Systems | Development | 185 |
| Organismal Systems | Digestive system | 336 |
| Organismal Systems | Aging | 291 |
| Organismal Systems | Endocrine system | 777 |
| Organismal Systems | Excretory system | 189 |
| Organismal Systems | Environmental adaptation | 115 |
| Organismal Systems | Sensory system | 157 |
| Organismal Systems | Immune system | 629 |
| Organismal Systems | Nervous system | 530 |
| Organismal Systems | Circulatory system | 250 |
| Total genes of Organismal Systems | | 3459 |
| Human Diseases | Cancers: Specific types | 295 |
| Human Diseases | Immune diseases | 160 |
| Human Diseases | Substance dependence | 184 |
| Human Diseases | Drug resistance: Antineoplastic | 268 |
| Human Diseases | Cancers: Overview | 1003 |
| Human Diseases | Infectious diseases: Viral | 760 |
| Human Diseases | Endocrine and metabolic diseases | 399 |
| Human Diseases | Infectious diseases: Bacterial | 528 |
| Human Diseases | Cardiovascular diseases | 270 |
| Human Diseases | Infectious diseases: Parasitic | 241 |
| Human Diseases | Neurodegenerative diseases | 612 |
| Total genes of Human Diseases |  | 4720 |
| Total genes mapped to KEGG pathways | | 33594 |

**Table S4. Unigenes of *M. alternatus* into COG terms**


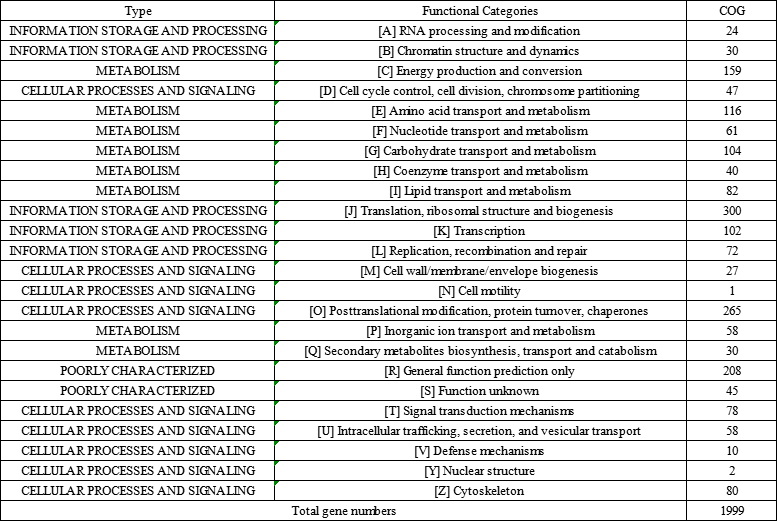


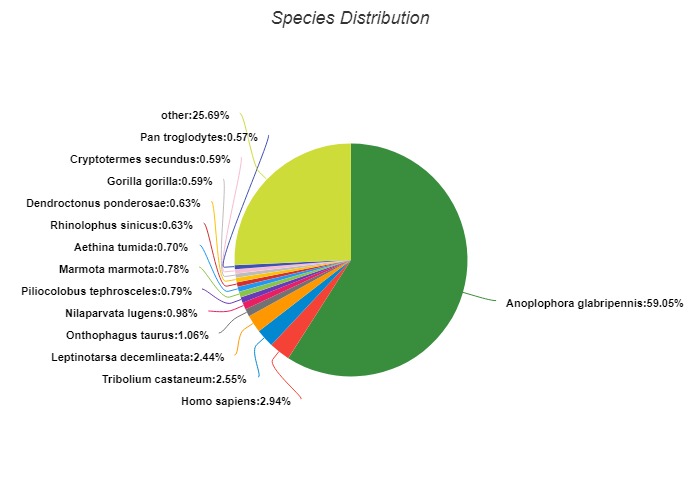


Figure S1. Transcriptome sequences of *Monochamus alternatus* matched with other species.

**
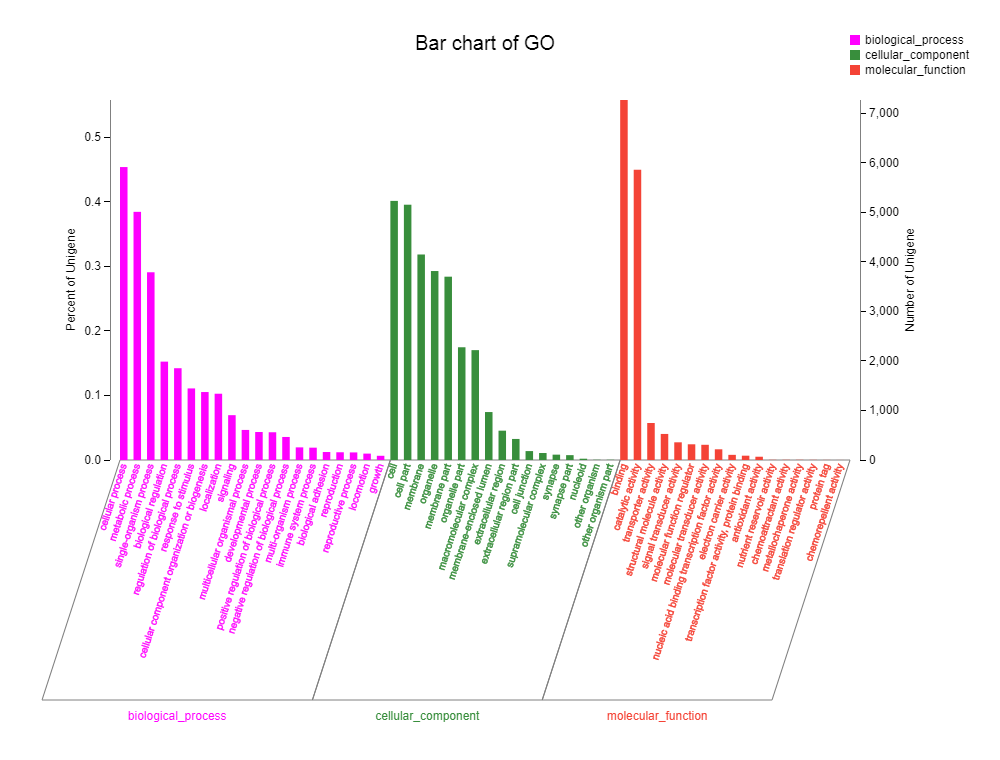
**

**Figure S2**. Clusters of Gene Ontology (GO) classification of unigenes


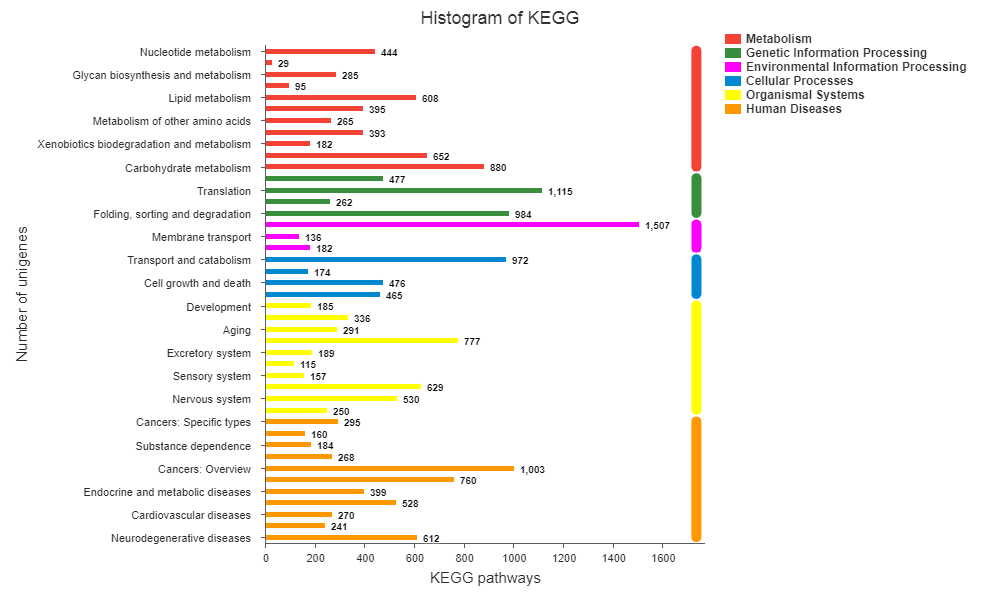
**Figure S3**. Clusters of Kyoto Encyclopedia of Genes and Genomes (KEGG) classification of unigenes


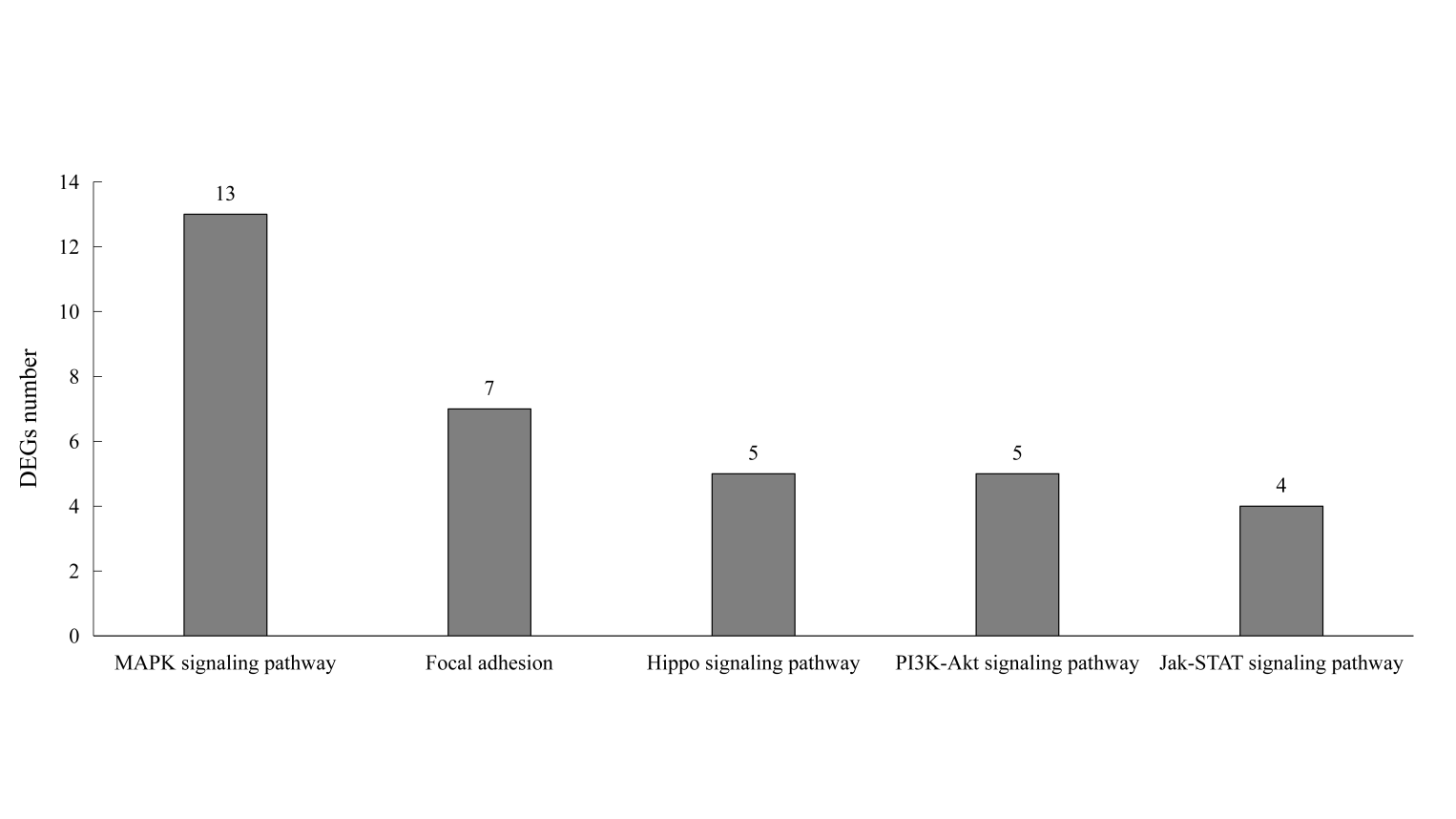


**Figure S4.** Number of upregulated genes associated with stress signal transduction pathways.
